# Indigenous approaches to perinatal mental health: a systematic review with critical interpretive synthesis

**DOI:** 10.1007/s00737-023-01310-7

**Published:** 2023-03-31

**Authors:** Cara Meredith, Christina McKerchar, Cameron Lacey

**Affiliations:** 1 Māori and Indigenous Health Innovation, University of Otago, Christchurch, New Zealand; 2 Population Health, University of Otago, Christchurch, New Zealand

**Keywords:** Perinatal mental health, Indigenous, Systematic review, Cultural approaches

## Abstract

Indigenous mothers and birthing parents experience significant inequities during the perinatal period, with mental health distress causing adverse outcomes for mothers/birthing parents and their infants. Limited literature is available to inform our understanding of solutions to these issues, with research primarily focusing on inequities. Our aim was to conduct a systematic review of Indigenous approaches to treatment of perinatal mental health illness. Following the PRISMA guidelines for systematic literature reviews, an electronic search of CINAHL, Medline, PubMed, Embase, APA PsycInfo, OVID Nursing, Scopus, Web of Science, and Google Scholar databases was conducted in January and February 2022 and repeated in June 2022. Twenty-seven studies were included in the final review. A critical interpretive synthesis informed our approach to the systematic review. The work of (Yamane and Helm J Prev 43:167–190, 2022) was drawn upon to differentiate studies and place within a cultural continuum framework. Across the 27 studies, the majority of participants were healthcare workers and other staff. Mothers, birthing parents, and their families were represented in small numbers. Outcomes of interest included a reduction in symptoms, a reduction in high-risk behaviours, and parental engagement/attachment of mothers/birthing parents with their babies. Interventions infrequently reported significant reductions in mental health symptoms, and many included studies focused on qualitative assessments of intervention acceptability or utility. Many studies focused on describing approaches to perinatal mental health distress or considered the perspectives and priorities of families and healthcare workers. More research and evaluation of Indigenous interventions for perinatal mental health illness is required. Future research should be designed to privilege the voices, perspectives, and experiences of Indigenous mothers, birthing parents, and their families. Researchers should ensure that any future studies should arise from the priorities of the Indigenous population being studied and be Indigenous-led and designed.

**Supplementary Information:**

The online version contains supplementary material available at 10.1007/s00737-023-01310-7.

## Introduction

Perinatal mental illness refers to a broad range of psychiatric disorders including mild depression and anxiety, stress-related disorders, postpartum psychosis, post-traumatic stress disorder, eating disorders, obsessive–compulsive disorders, and anxiety disorders. Disorders present prior to pregnancy, along with those that emerge during pregnancy or the postpartum period, are all considered to be perinatal mental illnesses (O’Hara et al. 2014; Black et al. 2019).

Perinatal mental health research has explored the impact of socio-environmental, psychological, and biological factors on disease and wellbeing across the life span. It is known that high levels of stress and associated mental distress can affect both immediate and long-term outcomes for parent and infant (O’Hara et al. 2014). In many countries, suicide is the leading cause of maternal death (Chin et al. 2022). Parents who are depressed often engage in high-risk behaviours and are less likely to seek or receive adequate healthcare during pregnancy and the postpartum period. Consequences include an increased risk for substance abuse, smoking, exposure to sexually transmitted and other diseases, family violence, poor nutrition, and reduced or absent support systems (O’Hara et al. 2014). These can result in obstetric complications, premature birth, and birth interventions leaving the infant at risk of lower Apgar scores, low birth weight and poor weight gain, poor breastfeeding outcomes, and increased rates of admission to neonatal intensive care units. In the long-term, affected infants are more likely to develop ongoing behavioural, psychological, developmental, and physical challenges (Bowen et al. 2014).

The internationally accepted timeframe for the perinatal period covers pregnancy through to the first year postpartum. However, the time frame of the perinatal period is debatable (O’Hara et al. 2014). Many services and interventions targeting parents and infants take a ‘life course approach’ and view perinatal mental health as fundamentally connected to the long-term wellbeing of infants. This encompasses the *First 1000* days of life and the widely accepted understanding that the period from pregnancy to age 3 years is when the foundation for health and wellbeing is established. (Maternal Care Action Group New Zealand (MCAGNZ) 2022; Linnér and Almgren 2020; Darling et al. 2020). Supporting the mental health of parents during this time is therefore critical. As such, the authors were interested in capturing the impact of services within the first 3 years of life.

Indigenous mothers/birthing parents experience disproportionate levels of mental health illness in the perinatal period compared to the general population (Black et al. 2019; Marriott et al. 2019; Owais et al. 2020). International studies and recent systematic reviews have highlighted disparities in both engagement in services and mental health outcomes for Indigenous populations during the perinatal period (Black et al. 2019; Owais et al. 2020; Tricklebank 2014).). To date, research has primarily focused on describing these inequities, with very little research exploring interventions and approaches to treatment of perinatal mental health that are effective and culturally appropriate for Indigenous populations. As Indigenous women/birthing parents experience a higher prevalence of perinatal mental health distress along with a higher birth rate than their non-Indigenous counterparts, (Bowen et al. 2014), there is a need for better understanding of interventions and approaches that target, and work for, this vulnerable population.

Pre-colonisation, Indigenous populations had systems of health and wellbeing attuned to the environment and concepts of collective and individual wellbeing. The process of colonisation imposed Euro-centric beliefs and systems of health, effectively impeding the self-determination of Indigenous people to manage their health and disrupting their systems of wellbeing (Kirmayer et al. 2003). This, combined with the broader experiences of colonisation such as language degradation, land theft, loss of resources and wealth, discrimination, and the consequent decreased access to the social determinants of health, has led to significant health inequities which persist today for these populations (McCalman et al. 2017; McKinley et al. 2021; Graham and Martin 2016).

Consistent with other areas of health, non-Indigenous models and approaches to treatment of perinatal mental health illness privilege non-Indigenous populations and world-views, perpetuating the inequities experienced by Indigenous peoples. International studies reiterate the need for culturally appropriate evaluation and treatment in the perinatal period for at-risk populations (Marriott et al. 2019). In response, some services and interventions have been adapted in an attempt to serve Indigenous groups more effectively. However, the effectiveness of these interventions in terms of perinatal mental health outcomes needs further exploration.

The aim of this systematic literature review is to identify and synthesise the characteristics of Indigenous approaches to perinatal mental health and wellbeing. The following research questions guided our review ‘What are the common characteristics of Indigenous approaches to Indigenous perinatal mental health and wellbeing?’ and ‘Are Indigenous led interventions that privilege Indigenous values, ways of knowing, and practices, effective for Indigenous mothers and birthing people experiencing perinatal mental health distress?’.

This review followed the approach as recommended by the United Nations (UN) to identify rather than define Indigenous peoples. The UN states: ‘Indigenous peoples are the holders of unique languages, knowledge systems and beliefs…their ancestral land has a fundamental importance for their collective physical and cultural survival as peoples. Indigenous peoples hold their own diverse concepts of development, based on their traditional values, visions, needs and priorities’ (UN, 2023).

In keeping with a commitment to use inclusive language, the term ‘birthing parents’ has been used alongside mothers and women throughout this paper.

## Methods

Following the Preferred Reporting Items for Systematic Reviews and Meta-analysis (PRISMA) guidelines (Page et al. 2021), we conducted a systematic review of the literature to search for studies examining Indigenous approaches to perinatal mental health. The aim of the review was to identify the characteristics of effective and appropriate approaches to Indigenous perinatal mental health and wellbeing. The PRISMA checklist is included in the supplementary information. A protocol for this review was registered on the international prospective register of systematic reviews (PROSPERO) (registration number: CRD42022346190*)*.*

### Eligibility criteria

Studies were eligible for inclusion if they met the following criteria: For population (P), studies included any Indigenous birthing parents/mothers experiencing (or at risk of experiencing) perinatal mental health distress. Intervention/phenomenon of interest (I) is any service, intervention, or model that identified a culturally responsive and/or Indigenous approach that has an impact on mental health and wellbeing during the perinatal period, up to 3 years. Studies exploring the perspectives of Indigenous parents and families on approaches to mental health distress and wellbeing in the perinatal period were also included. For outcomes (O), studies were included if they identified outcomes related to perinatal mental health and wellbeing (such as symptom reduction) and/or parental-child attachment for Indigenous families. Studies that did not include an Indigenous analysis were excluded. Indigenous analysis engages with Indigenous persons as investigators or partners to extend knowledge significant for those communities. Studies related to mental health beyond the defined perinatal period were also excluded. Only peer-reviewed published studies were included. Qualitative and quantitative studies were included. Inclusion and exclusion criteria are described in full in Table 1.

**Table 1:** Inclusion/exclusion criteria

PIO	Inclusion criteria	Exclusion criteria
Population	Indigenous populations Parents/mothers experiencing (or at risk of experiencing) mental health distress/illness in the defined perinatal period (pregnancy—3 years postpartum or period inclusive of the perinatal period)	Studies examining non-Indigenous parents/mothers only
Intervention	Any service, intervention, or model that identifies a culturally responsive and/or Indigenous approach that has an impact on mental health and wellbeing during the perinatal period	Any programme or service that does not specify an Indigenous/cultural response Interventions related to perinatal mental health screening Studies related to pregnancy and childbirth only Studies related to alcohol and other drugs (AOD)/substance abuse in the perinatal period Peer support interventions Research that does not include an Indigenous analysis
Outcome	Any related to perinatal mental health and wellbeing Outcomes related to maternal-child attachment	Outcomes related to parents/mothers beyond the defined perinatal period

### Literature search

The following databases were searched: CINAHL, Medline, PubMed, Embase, APA PsycInfo, OVID Nursing, Scopus, Web of Science, and Google Scholar. Search terms were structured around perinatal mental health, Indigenous populations, and Indigenous interventions/approaches (see Table 2). No restrictions were applied to study settings or publication dates, and only English language peer-reviewed articles were included. The literature search took place in January and February 2022 and repeated to check for new publications in June 2022. The complete search strategy for the review is included in the supplementary data (Online Resource). The database search was complemented by handsearching the reference lists of included articles and related systematic reviews.

**Table 2:** Search terms

	Search strategy
Perinatal mental health	Perinatal mental health/postnatal period/postpartum depression/perinatal anxiety/mothers/pregnancy/perinatal period AND mental health/maternal mental health
Indigenous populations	Indigenous populations/Māori/First Nations/Aboriginal/Alaskan Natives/Hawaiian Natives/Pacific/Pasifika/Polynesian/Torres Strait Island*/Indigenous Peoples/Native Americans/Canadian Natives/Indigenous Canadians
Indigenous interventions	indigenous services/Cultural Sensitivity/indigenous workers/kaupapa Māori/cultural responsiveness
Indigenous approaches	Rongoā/Toanga Pūoro/Ceremony/Two eyed seeing/Medicine wheel/spirituality/Elders/kaumātua/aunt*/Traditional medicine/customary

Duplicates were identified and removed using Endnote. Title/abstract screening was completed by (first author) and discussed and refined with co-authors. Full text screening was completed initially by (first author) and then independently reviewed by (co-authors). Any disagreements were discussed by all three authors and consensus met.

### Data extraction and analysis

Data extraction and analysis was carried out by first author and discussed with co-authors who have expertise in Indigenous health research. Data items from the studies were extracted and hand-coded in Excel format. Publication date, country and region, Indigenous population, sample size, age range of participants and age range of infant (to ensure study remained within defined perinatal period), setting, study aims and design, method of data collection, analysis, and outcomes were collated. The following data was then extracted and collated: type of intervention, characteristics of the intervention, and cultural characteristics identified within each study. Studies were further organised into a framework identified by Yamane and Helm (2022) that situates interventions on a cultural continuum, identifying approaches that are either culturally adapted, culturally grounded, or promote a ‘culture as health’ approach.

### Quality assessment

In view of the inequities experienced by Indigenous populations in relation to perinatal mental health, we were interested in the responsiveness of the research to the Indigenous populations being studied. We utilised the CONSolIDated criTEria (CONSIDER) statement (Huria et al. 2019) to assess whether studies included equitable and ethical health research practices involving Indigenous populations. The quality of these studies in relation to Indigenous priorities and the advancement of Indigenous health was also assessed using the CONSIDER tool (Meechan and Brewer 2021; Wright et al. 2022). Each of the 17 statements from the CONSIDER criteria was rated (yes (score of 1) or no (0 score)) to provide a total score out of a possible 17.

### Data synthesis

(i) ***Critical interpretive synthesis***

A critical interpretive synthesis (CIS) informed our approach to the systematic review. This approach was used to synthesise both the qualitative and quantitative research, allowing for the generation of themes and identifying the characteristics of Indigenous approaches to treatment of perinatal mental health illness and distress. CIS integrates evidence from across studies to form a conceptual framework that can be applied to clinical practice and inform future research (Dixon-Woods et al. 2006). Synthesis involved categorising approaches or ‘characteristics’ commonly identified across all studies.

(ii) ***Cultural continuum***

According to the characteristics identified, a cultural continuum framework was used to distinguish whether the included studies were *culturally adapted*, *culturally grounded*, or promoted a *culture as health* model. These three interpretations draw upon the work of Yamane and Helm (2022). Cultural adaptation is defined as a systematic modification of an evidence-based intervention or programme and considers aspects such as language and culture to become more congruent with the cultural context of the recipient of care (Bernal et al. 2009). Adaptations may be ‘surface level’ such as using or changing imagery, language, or music to reflect the population being targeted. ‘Deep level’ adaptations integrate cultural, social, environmental, and spiritual elements (Wiltsey Stirman et al. 2019). *Culturally grounded* interventions and programs are designed and led by Indigenous peoples, utilise Indigenous ways of knowing, and embed Indigenous values and beliefs. *Culture as health* progresses this concept and privileges Indigenous ways of knowing, doing, and being, over non-Indigenous approaches, and is defined by the inclusion of four paradigms: Indigenous ways of knowing, Indigenous cultural practices, place-based/sacred sites, and Indigenous spirituality (Yamane and Helm 2022). *Culturally grounded* interventions are more likely to incorporate non-Indigenous approaches than the c*ulture as health* approaches.

## Results

### Study selection

**Fig. 1:**
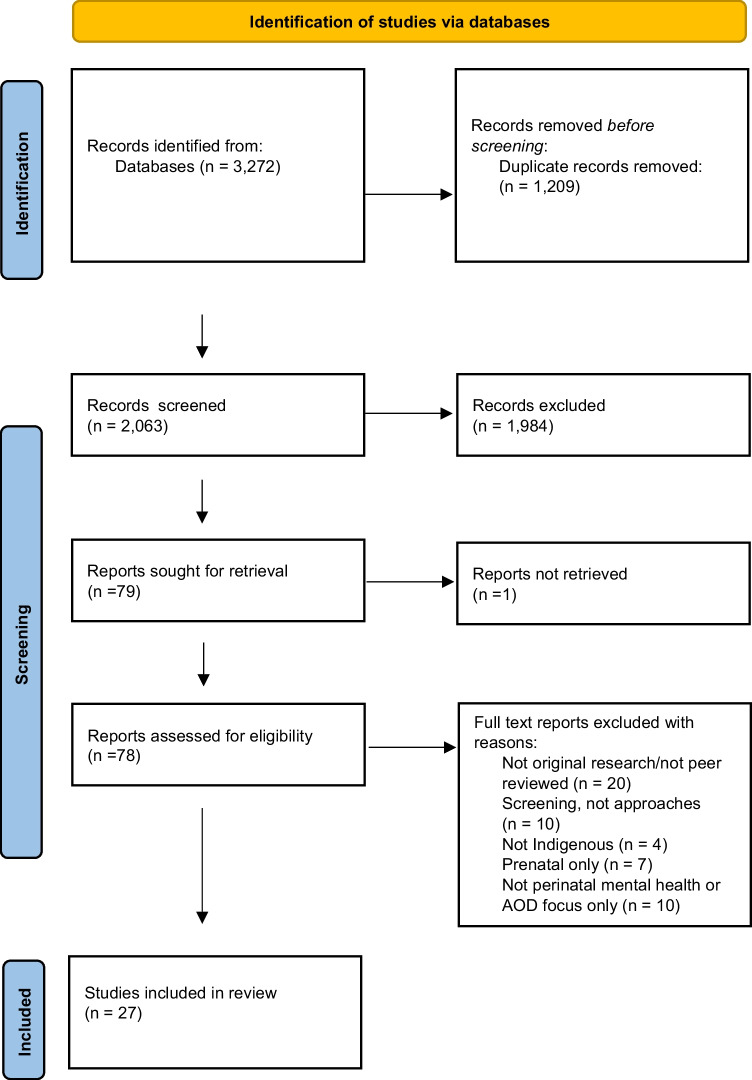
PRISMA flowchart

A total of 3272 articles resulted from our search of the databases. After removing duplicates, 2063 results were screened. Seventy-eight results were eligible for full-text screening. Figure 1 outlines the screening process (PRISMA flowchart) and reasons for exclusion. A total of 27 studies were eligible for inclusion.

### Study characteristics

The included studies were published between 2006 and 2022. Included studies were conducted across five countries: USA (*n* = 10), Australia (*n* = 8), Canada (*n* = 4), Aotearoa/NZ (*n* = 3), and Guatemala (*n* = 2). Table 2 describes specific characteristics of the 27 studies included in this review.

The interventions evaluated within the included studies either focused on mental health in the perinatal period or included mental health as part of a wider examination of perinatal health. Interventions evaluated were parenting programmes (*n* = 8); health programmes (*n* = 6); mental health-focused interventions (*n* = 5); cultural interventions (*n* = 2); family support (*n* = 1); and an engagement tool (*n* = 1). Cultural interventions address health and wellbeing holistically, focusing on a balance within health incorporating mind–body, spirituality, culture, language, community, and traditions (Bowen et al. 2014). Engagement tools encourage participation and provide an enticing way of supporting communities to engage in an aspect of healthcare. The remaining studies provided qualitative data on the perspectives of Indigenous peoples on approaches to perinatal mental health.

### Participants

Across the 27 studies, a total of 122 birthing parents and family members and 284 healthcare workers or other staff members were included. Of the studies that interviewed recipients of care only, the number of interviewed participants ranged from 14 to 19 in total. Of the healthcare workers and other non-family member participants interviewed, 46% were identified as Indigenous, and 27% were non-Indigenous. Ethnicity of the remaining 27% was either unclear or not stated.

### Study outcomes

Outcomes of interest included a reduction in symptoms, a reduction in high-risk behaviours, and parental engagement/attachment of mothers/birthing parents with their babies. The reporting of outcomes varied across all study types, with the majority of studies not reporting on specific outcomes (see Table 3). These studies were descriptive or designed to inform approaches to perinatal mental health distress and illness in Indigenous populations. *Culturally adapted* studies were most likely to report on outcomes (63%). Of the *culturally grounded* studies 33% reported on outcomes. No outcomes were reported or measured within the studies identified as being *culture as health.*

**Table 3:** Study characteristics

First author (year)	Country (region)	Study type	Intervention/approach	Aims	Sample size (# Indigenous)	Participants (Indigenous group, type)	Measures and outcomes
Ginsburg et al. (2012)	USA (Arizona)	RCT	Parenting program—*Living in Harmony*	To evaluate the feasibility of a depression prevention program for American Indian adolescents and young adults in the perinatal period	47 (47)	White Mountain Apache Pregnant women/parents (28-week gestation–24 weeks postpartum)	**Measures used:** Edinburgh Postnatal Depression Scale (EPDS) Center for Epidemiologic Studies Depression Scale (CES-D) Diagnostic Interview Schedule for Children (DISC) Social Support Index(SSI) Global Assessment Scale for Children (CGAS) **Outcomes:** Both intervention arms showed similar rates of symptom reduction
Walkup et al. (2009)	USA (Arizona)	RCT	Parenting program—*Family Spirit*	To evaluate the efficacy of a paraprofessional-delivered, home-visiting intervention among young, reservation-based American Indian mothers on parenting knowledge, involvement, and maternal and infant outcomes	167 (167)	Navajo and White Mountain Apache Pregnant women/parents 28-week gestation–12 months postpartum	**Measures used:** Parenting knowledge self-report test Parent involvement self-report test HOME (Home Observation for Measurement of the Environment) Infant Toddler Social Emotional Assessment (ITSEA) CES-D Substance Use self-reported Social Support self-report test Parenting Stress Index **Outcomes:** No differences between groups were found for parental stress, depression or substance abuse or maternal involvement
Zarnowiecki et al. (2018)	Australia (central)	Mixed methods study (quantitative data from parents records and qualitative data from staff)	The Australian Nurse-Family Partnership Program (ANFPP)	To describe the complexity of Program clients in the Central Australian family partnership program, understand how client complexity affects program delivery and the implications for desirable program modification	Parents: 276 (not stated implied 276 Indigenous) Staff 11 (5)	Australian Aboriginal women/parents and staff members	**Measures used:** Demographic details form, Maternal Health Assessment Form, Relationship Assessment form (Family Violence) Adversity checklist of vulnerabilities (inc poor mental health) EPDS **Outcomes:** No post intervention outcomes measured
Barlow et al. (2015)	USA (Southwest)	RCT	Parenting program—*Family Spirit*	To report 36-month outcomes of the paraprofessional-delivered Family Spirit home-visiting intervention for American Indian teen mothers and children	322 (322)	White Mountain Apache and Navajo Adolescent women/parents 32-week gestation–36 months postpartum	**Measures used:** CES-D **Outcomes:** Parents in the intervention group showed fewer depressive symptoms (effect size = 0.16)
Ward et al. (2022)	USA (South Dakota)	Qualitative	Perinatal depression prevention intervention	To describe the development of cultural adaptations to Mothers and Babies, an evidence-based perinatal depression prevention intervention	14 (14)	Lakota Mothers/birthing parents and elders Newborn–unclear to what age	**Measures used:** None **Outcomes:** No outcomes measured
Hiratsuka et al. (2018)	USA (Washington State, New Mexico, Alaska)	Case studies	Parenting programs aimed at birthing and early parenting period	To describe the steps that four Tribal MIECHV Programs took to assess community needs, select a home-visiting model, and culturally adapt the model for use in AI/AN communities	Not stated/N/A	American Indian, Alaskan Natives (not further defined) Mothers/birthing parents and families Pregnancy–3 years	**Measures used:** None **Outcomes:** No outcomes measured
Barlow et al. (2006)	USA (New Mexico and Arizona)	RCT	Home visiting intervention	To assess the impact of a paraprofessional-delivered home-visiting intervention to promote child care knowledge, skills, and involvement among pregnant American Indian adolescents	53 (53)	Apache and Navajo adolescent mothers/birthing parents 28-week gestation–6 months postpartum	**Measures used:** Child care knowledge and skills test scores Maternal self-reports **Outcomes:** No between-group differences for psychological and behavioural risk scores
Penehira and Doherty (2013)	Aotearoa NZ (South Auckland)	Qualitative	Parenting program—Mellow Parenting	The aim of this pilot study was to evaluate the acceptability and effectiveness of *Hoki ki te Rito* (HKTR)/Mellow Parenting program, for Māori mothers in South Auckland, New Zealand	Not stated	NZ Māori mothers/birthing parents and grandmothers/parents Two staff members	**Measures used:** Mother’s self-reported competence, stress, and wellbeing post **Outcomes:** Self-reported positive outcomes on parental stress and wellbeing noted
Mullany et al. (2012)	USA (Southwest)	RCT	Parenting program—*Family Spirit*	To evaluate the impact of the paraprofessional-delivered ‘Family Spirit’ home-visiting intervention to reduce health and behavioural risks for American Indian teen mothers and their children	322 (322)	American Indian, Alaskan Natives (not further defined) mothers/birthing parents 28–32-week gestation–36 months postpartum	**Measures used:** *(each collected at 9 time points throughout the study period)* Parental competence outcomes (Parent Knowledge Test; Parental Locus of Control (PLOC); Home Safety Assessment; HOME; Supplement to HOME for Impoverished Families (SHIF); Adult Adolescent Parenting Inventory (AAPI-2); Parental Sense of Competence (PSOC); Parenting Stress Index–Short Form (PSI-SF) Child psychosocial and behavioural outcomes: ITSEA; Ages and Stages Questionnaire (ASQ); Child Behavior Checklist (CBCL); Medical Record Review Maternal psychosocial and behavioural outcomes: Achenbach System Empirically Based Assessment: Youth Self Report (ASEBA); CES-D; Voices of Indian Teens (VOIT): Cultural identity; Voices of Indian Teens (VOIT): Alcohol; Voices of Indian Teens (VOIT): Drugs, Ideas, Thoughts and Happenings; Audio Computer Assisted Self- Interview (ACASI); Problem Oriented Screening Instrument for Teens (POSIT); Medical Record Review **Outcomes:** Baseline results collected, no post-intervention outcomes reported within this study
Campbell et al. (2018)	Australia (Cape York, Queensland)	Qualitative	Baby One Program	To present a study of implementation of the Baby One Program (BOP). The BOP was designed as a family-centred, Indigenous Healthworker-led, home-visiting model of care focused on promoting family health to give children the best start to life	48 (40)	Aboriginal and Torres Strait Islander Gestation–2 years and 10 months	**Measures used:** None **Outcomes:** Positive outcomes reported: reduction in substance abuse, increased engagement, improved generalised wellbeing
Kohrt et al. (2022)	Guatemala (Sololā)	Mixed methods	Task-sharing program for perinatal depression	To describe the process of adapting the Thinking Healthy Programme (THP), a task-sharing intervention that targets perinatal depression, for use within a community health organization serving indigenous, Tz'utujil Mayan families in the Solola region of Guatemala	25 (15)	Tz'utujil Mayan Mothers / Birthing parents New-born– not stated	**Measures used:** None **Outcomes:** No outcomes measured
McCalman et al. (2015)	Australia (Queensland)	Qualitative	Baby Basket program	To theorise the process of implementing an Indigenous Australian maternal and child health program	28 (28)	Aboriginal and Torres Strait Islander Pregnant women/birthing parents and healthcare workers Pregnancy/newborn—not stated	**Measures used:** None **Outcomes:** Positive outcomes reported included increased engagement, some reduction in high-risk behaviours
Chomat et al. (2019)	Guatemala (San Juan Ostuncalco)	Mixed methods	Women’s Circle	To test acceptability, feasibility and impact of a co-designed group psychosocial intervention (Women’s Circles) in a population with significant need but no access to mental health services	155 (155)	Mam and K'iche Pregnant women/birthing parents and healthcare workers newborn—not stated	**Measures used:** Hopkins Symptom Checklist-25 (HSCL-25) Mental Health Continuum Short Form (MHC-SF) UNICEF Multiple Indicator Cluster Survey Early Child Development **Outcomes:** Improved wellbeing scores were higher among intervention group
Smith et al. (2007)	Canada (region not stated)	Qualitative	Indigenous led perinatal care	To describe community-based stakeholders’ views of how safe and responsive care ‘makes a difference’ to health and wellbeing for pregnant and parenting Aboriginal people	57 (35)	Aboriginal-Canadian (not specified) Leaders, providers, and community members	**Measures used:** None **Outcomes:** Improved engagement, reduction in high-risk behaviours, and some improvement in attachment
Ussher et al. (2016)	Australia (Sydney, NSW)	Qualitative	Early intervention program for Aboriginal mothers/birthing parents	To examine Aboriginal women’s subjective experiences and constructions of motherhood in the context of early intervention programs and the perceived impact of such programs	19 (10)	Australian Aboriginal Mothers/birthing parents (10)) healthcare workers (9) 3 months–4 years	**Measures used:** None **Outcomes:** Positive outcomes reported included increased confidence, resilience, improved attachment
Barlow et al. (2013)	USA (Arizona)	RCT	Parenting program—*Family Spirit*	To examine the effectiveness of Family Spirit, a paraprofessional-delivered, home-visiting pregnancy and early childhood intervention, in improving American Indian teen mothers’ parenting outcomes and mothers’ and children’s emotional and behavioural functioning 12 months postpartum	322 (322)	American Indian, Alaskan Natives (not further defined) mothers/birthing parents 28–32-week gestation–12 months postpartum	**Measures used: (***collected at 4 time points during the study period)* Parenting knowledge; PLOC scale; HOME scale; ITSEA **Outcomes:** No between-group differences noted for substance abuse, depression scores (CES-D) measured/reported at baseline only
Dietsch et al. (2011)	Australia (NSW)	Qualitative	Family support	To explore the significance for Aboriginal women when they are denied the support of kin around the time of birth but have that support re-established postnatally	5 (5)	Australian Aboriginal Mothers/birthing parents and midwives	**Measures used:** EPDS **Outcomes:** Enhanced wellbeing and possible prevention of mental ill health reported. Low EPDS scores
Lowell et al. (2015)	Australia (Northern Territory)	Qualitative	Family support	An evaluation to identify enabling factors and barriers to successful implementation of the Program, and to identify potential pathways for future development. In this paper, we focus on the evaluation findings related specifically to the role of Aboriginal cultural knowledge and practice within the Program	76 (unclear)	Aboriginal and Torres Strait Islander Healthcare workers and community Newborn—unclear to what age	**Measures used:** None **Outcomes:** No outcomes measured
Smith et al. (2006)	Canada (British Colombia)	Qualitative	Indigenous led perinatal care	To investigate 2 Aboriginal organisations’ experiences improving care for pregnant and parenting Aboriginal people	73 (44)	First Nations (not further specified) providers, community leaders, community members Pregnancy—early parenting	**Measures used:** None **Outcomes:** No outcomes measured or reported
Blanchet-Cohen et al. (2021)	Canada (Quebec)	Qualitative	Abinodjic primary healthcare program	To describe the emergence and relevance of a model of perinatal care where parental experiences, healthy lifestyles, support networks, and cultural knowledge are four interdependent areas of intervention that support children’s wellbeing, in the context of culturally safe services and approaches	15 (15)	Anishinaabe Mothers/birthing parents Pregnancy—2 years	**Measures used:** None **Outcomes:** Positive outcomes reported included increased engagement, strengthened relationships, reduced isolation
Kandasamy et al. (2017)	Canada (Ontario)	Qualitative	Family perspectives on perinatal period	To investigate elder Indigenous women’s perceptions around optimal perinatal health	18 (18)	Six Nations (Mohawk, Seneca, Oneida, Cayuga, Onondaga, Tuscarora) Grandmothers/parents	**Measures used:** None **Outcomes:** No outcomes measured
Reid et al. (2021)	Australia	Qualitative	Healing the past, nurturing the future project	This research aims to identify and refine culturally appropriate support strategies for Aboriginal and Torres Strait Islander parents experiencing complex trauma	54 (21)	Aboriginal and Torres Strait Islander Professionals, parents, and community members with an interest in Indigenous perinatal and maternal health and wellbeing	**Measures used:** None **Outcomes:** No outcomes measured
Abbott et al. (2022)	USA (South Dakota)	Qualitative	Talking Circles	To understand the primary psychosocial stressors that are major contributors to pregnancy and parenting stress that American Indian (AI) women face during pregnancy and to identify mechanisms of resilience	14 (14)	Lakota Mothers/birthing parents and grandmothers/parents	**Measures used:** None **Outcomes:** No outcomes measured
Chamberlain et al. (2021)	Australia (Alice Springs, Adelaide and Melbourne)	Qualitative	Healing the past, nurturing the future project—family perspectives	To co-design perinatal awareness, recognition, assessment strategies for Aboriginal and Torres Strait Islander parents experiencing complex trauma and support	17 (17)	Aboriginal and Torres Strait Islander Mothers/birthing parents and partners	**Measures used:** None **Outcomes:** No outcomes measured
Ware et al. (2015)	Aotearoa NZ	Qualitative	Kaupapa Māori parenting approaches	To explore the challenges of being Mäori and a young parent, the potential of positive representations of reproduction and caregiving from Te Ao Mäori (the Māori world) and navigation to a positive identity	19 (19)	NZ Māori mothers/birthing parents and partners 2 months to 5 years	**Measures used:** None **Outcomes:** No outcomes measured
Berryman et al. (2022)	Aotearoa NZ	Qualitative	Māori approaches to pregnancy, birth, and the postnatal period	To explore and present ancestral knowledge capable of indigenising and decolonising current constructs about conception, pregnancy, birth and infancy	15 (15)	NZ Māori mothers/birthing parents, elders, and family Perinatal period—not further defined	**Measures used:** None **Outcomes:** No outcomes measured
James et al. (2021)	USA (Washington State)	Qualitative	Cradle board making, talking circles—intervention aimed at perinatal period	To present a case study of a culturally based educational intervention on American Indian, Alaskan Natives maternal and child health	2 (2)	American Indian, Alaskan Natives (not further defined) Healthcare workers	**Measures used:** None **Outcomes:** No outcomes measured

### Quality assessment

Using the CONSIDER statement criteria, studies were determined to have a range of scores from 0 to 12 (out of a possible 17). Common areas of omission (resulting in lower scores) for most studies were around governance or partnership agreements, suggesting an absence of Indigenous leadership. For example, only half of the studies included in this review described how the research emerged from the priorities identified by Indigenous stakeholders (full results of quality assessment are found in the supplementary information (Online Resource). The total scores of each study are found in Table 4

**Table 4:** Cultural characteristics—cultural continuum

Study	Intervention type	Cultural continuum	Bicultural approach	Indigenous ways of knowing	Indigenous cultural practices	Place based/sacred sites	Indigenous spirituality	Cultural values/beliefs	Designed from ground up	Consider score (out of possible 17)
Ginsburg et al. (2012)	Living in Harmony (LIH)	Adapted								2
Walkup et al. (2009)	Family Spirit	Adapted	*							3
Zarnowiecki et al. (2018)	The Australia Nurse-Family Partnership	Adapted								0
Barlow et al. (2015)	Family Spirit	Adapted	*							5
Ward et al. (2022)	Mothers & Babies Intervention	Adapted	*							11
Hiratsuka et al. (2018)	Culturally adapted home visiting programs	Adapted	*							0
Barlow et al. (2006)	Home visiting intervention	Adapted								1
Penehira and Doherty (2013)	Mellow Parenting	Adapted	*							7
Mullany et al. (2012)	Family Spirit	Adapted								11
Campbell et al. (2018)	Baby One Program	Adapted	*							4
Kohrt et al. (2022)	Task-sharing program for perinatal depression	Adapted								3
McCalman et al. (2015)	Baby Basket program	Adapted								3
Chomat et al. (2019)	Women’s Circle	Adapted								6
Smith et al. (2007)	Indigenous led perinatal care	Adapted								5
Ussher et al. (2016)	Early intervention program	Adapted								0
Barlow et al. (2013)	Family Spirit	Adapted								1
Dietsch et al. (2011)	Family support	Grounded		*		*		*	*	2
Lowell et al. (2015)	Strong Women, Strong Babies, Strong Culture	Grounded	*	*	*	*	?	*	*	6
Smith et al. (2006)	Indigenous led perinatal care	Grounded	*	*	*	?	*	*	*	6
Blanchet-Cohen et al. (2021)	Abinodjic primary healthcare program	Grounded	*	*	*	?	?	*	*	10
Kandasamy et al. (2017)	Family Voice	Grounded	*	*			*	*	*	12
Reid et al. (2021)	Healing the past, nurturing the future project	Grounded	*	*	*			*	*	10
Abbott et al. (2022)	Talking Circles	Culture as health	*	*	*	*	*	*	*	7
Chamberlain et al. (2021)	Family Voice	Culture as health	*	*	*	*	*	*	*	11
Ware et al. (2015)	KM parenting approaches	Culture as health	*	*	*	*	*	*	*	8
Berryman et al. (2022)	Māori approaches to pregnancy and parenting	Culture as health	*	*	*	*	*	*	*	9
James et al. (2021)	Cradle board making, talking circles	Culture as health	*	*	*	*	*	*	*	5

### Cultural continuum

Of the 27 included studies, 16 were identified to be *culturally adapted*, six *culturally grounded*, and five studies met all of the criteria to be distinguished as *culture as health* approaches (see Table 4).

Dominant characteristics of *culturally adapted* approaches included interventions such as counselling, psychoeducation, and problem solving skills (see S1 table in supplementary information, Online Resource). *Culturally adapted* approaches also advocated for Indigenous workers who were familiar with the native language and customs, promoting a culturally competent workforce. These characteristics were shared across the *culturally grounded* and *culture as health* studies. In this review, *culturally grounded* and *culture as health* studies were found to incorporate additional characteristics such as Indigenous practices, values, and spirituality. (Table 4 outlines the cultural characteristics of the included studies and places each study on the cultural continuum as defined by Yamane and Helm (2022)).

The identified characteristics were synthesised into six key themes: skills and education, support systems, relationships, Indigenous self-determination, Indigenous customs and practices, and Indigenous identity.

i) ***Skills and education***

Behavioural skills were identified by 74% of studies alongside problem solving skills (78%). Psychoeducation was identified as a key characteristic in 45% of the studies. This theme was dominant in the *culturally adapted* and *culturally grounded* studies and did not feature strongly in the *culture as health* defined studies.

ii) ***Support systems***

Social supports featured frequently across all studies with 85% of studies highlighting this as a key characteristic. Other forms of support systems included family, therapy or counselling, future planning or goal setting, and addressing the social determinants of health.

iii) ***Relationships***

Relationship building and the development of trust were identified as a key characteristic of 96% of the included studies. Relationships encompassed home visiting, trust building, and trauma informed approaches. Relationships were identified as being developed over time. *Culturally adapted* studies generally had a recommended or pre-set number of contacts with clients.

iv) ***Indigenous self determination***

Indigenous self-determination encapsulated approaches such as Indigenous leadership, Indigenous healthcare workers, interventions being Indigenously led and designed, involvement of elders, and the expression of self-determination of the Indigenous groups involved in the studies. These approaches all featured strongly in the *culturally grounded* and *culture as health* studies. *Culturally adapted* studies primarily featured the use of Indigenous workers and did not generally identify elders, self-determination or Indigenous design, and leadership as characteristics.

xxii) ***Indigenous customs and practices***

Family, Indigenous practices, and intergenerational knowledge sharing were all characteristics identified under the theme of Indigenous customs and practices. The role of family was identified as an important approach to perinatal mental health in 85% of the studies. Where characteristics under this theme were not identified, this was generally within the *culturally adapted* studies.

vi) ***Indigenous identity***

Characteristics such as connection to cultural identity, use of Native language, and connection to Indigenous spirituality were all determined to be categorised under the theme of Indigenous identity. Connection to cultural identity and spirituality were common in both *culturally grounded* and *culture as health* studies and were less likely to be found in the *culturally adapted* studies.

## Discussion

This is the first systematic review, to our knowledge, that examines Indigenous approaches to treatment of perinatal mental health illness or distress. Previous systematic reviews in this area have focused on outcomes, screening, and disparities for Indigenous populations experiencing perinatal mental health distress (Black et al. 2019; Marriott et al. 2019; Owais et al. 2020). Despite no restrictions placed on dates of publication, only 27 studies were identified that fit the inclusion criteria. The earliest studies were found in 2006. Interest and publication rate in this area appears to be increasing rapidly with almost half of the included studies being published in the last 5 years. Despite Indigenous peoples inhabiting more than 90 countries globally (United Nations Statistical division State of the World’s indigenous peoples 2009), the majority of the included studies were conducted in Australia, Aotearoa, Canada, and the USA. Colonisation and Euro-centric health systems are a shared experience of these countries, which may account for the focus on Indigenous perinatal mental healthcare in these countries and the relative availability of studies.

### Characteristics of approaches and the cultural continuum

The themes identified within this review are consistent with other literature exploring perinatal mental health. Social support is identified in several studies exploring perinatal mental health distress in general populations and is found to be protective, reducing the impact of stress during pregnancy, and mitigating the impact of stress during the postnatal period (Lavender et al 2016; Inekwe & Lee 2022; Shorey et al 2015; Li et al 2021). Perinatal interventions that strengthen social support are therefore recommended for both preventing and treating mental health distress. Behavioural and coping skills were also identified as key characteristics within the skills and education theme, aligning with other literature in this area (Lavender et al. 2016). The third theme of relationships aligns with both the Indigenous literature (Flaminio et al. 2020) and non-Indigenous literature. In Indigenous populations, relationships included close family and wider kinship groups, along with health practitioners. In non-Indigenous literature, these relationships were found in the form of continuity of care models of perinatal care, parenting groups, or peer support programs (Alderdice et al. 2013; Barlow et al. 2012; McLeish and Redshaw 2017).

The characteristics identified within the *culturally grounded* and *culture as health* studies within this review encompassed three interconnected domains: Indigenous self-determination, Indigenous identity, and Indigenous customs and practices. These domains are also considered to be cultural determinants of health (Verbunt et al. 2021), and it is therefore unsurprising that these themes were revealed within this review.

Qualities of self-determination were frequently discussed within the qualitative studies of this review. Indigenous self-determination is discussed in wider health literature as being key to Indigenous health and wellbeing. Self-determination is the process by which a person has agency and control over their own life and choices. It is fundamental to addressing power imbalances and ensures Indigenous peoples are involved in every layer of the decision-making process (Verbunt et al. 2021). Experiences that diminish, demean, or disempower an individual, particularly in relation to their cultural identity (Gerlach 2012), impact a person’s ability to be self-determining and contribute to poor health outcomes. These culturally unsafe interactions are often a feature when health services are controlled by a system that reflects the dominant science-based biomedical model of care and do not consider or acknowledge cultural or social determinants of health and wellbeing (Haynes et al. 2014).

Indigenous self-determination is innately linked with Indigenous leadership, and both are essential for positive health outcomes (Gallagher 2019). Elders are an inherent feature of Indigenous leadership and are identified as a key characteristic in some of the studies in this review. Elders instil values by way of example, view themselves as a bridge between the past and the future transmitting knowledge to future generations, and have a broader view of wellbeing and health to encompass spiritual realms as much as physical (Muru-Lanning et al. 2021; Verbunt et al. 2021).

Elders are also part of the wider family support and kinship ties that were a strong feature in this review, connecting to the theme of relationships. Kinship ties are viewed as an essential element of health as these relationships and connections have the ability to provide emotional and spiritual support, restoring balance during times of stress and illness (Willing et al. 2020). Kinship ties are a fundamental element of Indigenous identity (Killsback 2019; Monchalin et al. 2020). Cultural identity is a theme that has also been highlighted in other literature as important in connection to health and wellbeing (Brown et al. 2021) alongside connection to spirituality, ancestors, and cultural practices (Verbunt et al. 2021). Traditional healing practices have been highlighted as important for Indigenous health (Asamoah 2022) with traditional ceremonies providing a culturally safe avenue for Indigenous peoples to express feelings and engage with each other, promoting social support and collective transformation (Graham and Martin 2016).

Within this review, the majority of the *culturally grounded* and *culture as health* studies advocated or utilised a bicultural approach. A bicultural approach acknowledges the cultural identity of Indigenous peoples and integrates both Indigenous and non-Indigenous knowledge and practices (Eketone and Walker 2015). Interventions within the included studies generally incorporated biomedical approaches to mental health such as psychoeducation and counselling, alongside cultural practices or the integration of Indigenous values. Within the qualitative studies, recipients of care recognised the value of both approaches.

Indigenous concepts such as Two-Eyed Seeing and *He awa whiria* align with this dual approach. Two-Eyed Seeing is a conceptual framework introduced by Mi’kmaw Elder Albert Marshall in the mid-2000s (Broadhead and Howard 2021) that promotes viewing the world with one eye grounded in Indigenous knowledge with the other eye grounded in non-Indigenous knowledge. *He awa whiria* or ‘braided river’ is another Indigenous metaphor arising from a Māori worldview that considers the relationship between Māori and non-Māori streams of knowledge and creates space for both to integrate (Cram et al. 2018). Integrating both knowledge utilises the strengths of both and provides a more holistic approach (Wright et al., 2022; Liebenberg et al. 2022; Asamoah 2022). Wider mental health research has argued that interventions for Indigenous people need to be holistic in their approach, encompassing emotional, spiritual, physical, and mental wellbeing as well as being congruent with Indigenous culture and focusing on both the social and cultural determinants of health (Graham and Martin 2016; Dudgeon and Bray 2018; Liebenberg et al. 2022).

Cultural determinants or ‘cultural modes’ (Kitayatna et al. 2007) include customs, language, and values. Cultural modes are fundamental to health and wellbeing as they are related to self-esteem and cultural identity. They offer protection against stress and anxiety and provide the context for how people navigate social and health needs (Subica and Uchida 2022). This protection may be disrupted when cultural trauma (such as loss of land or language or any trauma affecting cultural identity) is experienced, impacting a person’s (or community’s) health and wellbeing (Subica and Uchida 2022).

The incorporation of cultural modes is supported within a bicultural model of care and can potentially mitigate the impact of cultural trauma. This approach also addresses the discord between the dominant biomedical system and the beliefs and perspectives of Indigenous peoples, promoting engagement with services (Sylliboy and Hovey 2020), Liebenberg et al. 2022). Re-engagement with Indigenous cultural and healing practices develops resilience and strength (Kopua et al. 2020) and can contribute to supporting better outcomes.

Improved outcomes in the perinatal period prevents the long-term negative health impacts on both parent and child, disrupting intergenerational cycles of negative impact on affected individuals and their families (Wilkinson et al. 2022). There is evidence that untreated perinatal mental health illness represents a heavy economic burden globally. Addressing this global issue would not only benefit the health and wellbeing of mothers/birthing parents and their families, but would also decrease the burden on health and social services, widely benefiting society as a whole. (Luca et al. 2020). In view of the health, social, justice, education, and economic disparities present for Indigenous populations globally, it is imperative that the perinatal wellbeing of Indigenous peoples be prioritised.

### Limitations

Limitations of this review are threefold. Firstly, the perinatal mental health of Indigenous people is relatively under researched, with literature primarily focused on disparities rather than efficacy of interventions. In particular, literature around Indigenous populations outside of Australia, New Zealand, the USA, and Canada was scarce. Secondly, both the paucity of literature and the heterogeneity of the studies made synthesis challenging and findings inconclusive. While psychotherapy and counselling were found to be common features of many studies, they were not described in detail, further limiting any attempts at analysis. Outcome measurements were inconsistent across studies, with some measurement tools validated for Indigenous populations, others not. Many studies did not measure or record outcomes.

Whilst *culturally grounded* and *culture as health* interventions are advocated for, there is minimal data and evidence for these types of interventions in the perinatal period. Much of the available literature focused on the description of programmes and approaches. Consequently, the perspectives of healthcare workers and the wider community dominated. These studies were included to ensure that the perspectives of parents and their families involved within these studies were not missed. However, the voices of those directly affected by perinatal mental health illness were diluted, compounding the inconsistency of available data. No studies examined the consistency between provider and patient views, and very few studies examined the interplay between provider and Indigenous pregnant/postpartum person on perinatal distress. The inclusion of the perspectives of birthing people affected by perinatal mental illness is of particular importance. This is to ensure that identified outcomes have relevance and meaning to the population involved (Kersting et al. 2022). Insights from recipients of care and their families can support a balanced and comprehensive assessment of outcomes and efficacy of treatment.

Finally, the CONSIDER quality assessment tool has identified that most of the literature is weak in terms of its ability to be inclusive of Indigenous people and cultural appropriateness of the research for these groups. This is consistent with the wider literature. A recent review of Indigenous methodologies used in maternity research revealed that less than 2% of articles described or reported all Indigenous research principles, and 71% of articles did not report on Indigenous people’s involvement (Patterson et al. 2022). Much Indigenous literature is unpublished, and this review may have been strengthened had it included grey literature such as reports and evaluations on Indigenous perinatal mental health initiatives.

## Conclusion

Approaches for Indigenous mothers, birthing parents, and their families experiencing perinatal mental health illness were identified in this review and highlighted bicultural and holistic approaches. The gaps within the research of Indigenous perinatal mental health have been identified: (1) The voices of Indigenous mothers, birthing parents, and their families are limited within the available literature. (2) Research about Indigenous programmes addressing mental health illness during the perinatal period is sparse. (3) There is a low number of peer-reviewed studies documenting outcomes and characteristics of Indigenous interventions. These gaps demonstrate the need for more research and evaluation of interventions. Of particular note, Aotearoa NZ had three studies within this review, and only one of these was related to an intervention. This paucity in available literature is consistent with other reviews exploring Indigenous interventions (Richer and Roddy 2022; Moses et al. 2022). Finally, important questions are raised around research practices and ensuring that research in this area arises from priorities identified by Indigenous communities, is Indigenous led, and has meaningful impact for the community being researched. Future research should be designed to privilege the voices, perspectives, and experiences of Indigenous mothers, birthing parents, and their families. Developing culturally appropriate services and programmes is crucial in order to address the immediate and long-term inequities faced by Indigenous families and communities. Research is required to inform effective and accessible interventions for Indigenous peoples experiencing mental health distress in the perinatal period.

## Supplementary Information

Below is the link to the electronic supplementary material.

Supplementary file1 (PDF 292 KB)

## Data Availability

The authors confirm that the data supporting the findings of this study are available within the article [and/or] its supplementary materials or are available on request from the corresponding author, [C.M].
