# Supplementary material for: Indigenous approaches to perinatal mental health: a systematic review with critical interpretive synthesis
Source: Arch Womens Ment Health. 2023 Mar 31;26(3):275–93. doi: 10.1007/s00737-023-01310-7 (PMC10191969; doi:10.1007/s00737-023-01310-7)
Supplement: Supplementary file 1 — Supplementary file1 (PDF 292 KB) [file 737_2023_1310_MOESM1_ESM.pdf]

Supplementary Material for "Indigenous Approaches to Perinatal Mental Health: A Systematic Review with Critical Interpretive Synthesis"

Contents

1) **PRISMA checklist**
2) **Full search strategy**
3) **CONSIDER results table**
4) **General characteristics of included studies**

APPENDIX 1: PRISMA checklist

Section and Topic	Item #	Checklist item	Location where item is reported
**TITLE**			
Title	1	Identify the report as a systematic review.	1
**ABSTRACT**			
Abstract	2	See the PRISMA 2020 for Abstracts checklist.	1
**INTRODUCTION**			
Rationale	3	Describe the rationale for the review in the context of existing knowledge.	1-2
Objectives	4	Provide an explicit statement of the objective(s) or question(s) the review addresses.	2
**METHODS**			
Eligibility criteria	5	Specify the inclusion and exclusion criteria for the review and how studies were grouped for the syntheses.	3
Information sources	6	Specify all databases, registers, websites, organisations, reference lists and other sources searched or consulted to identify studies. Specify the date when each source was last searched or consulted.	3
Search strategy	7	Present the full search strategies for all databases, registers and websites, including any filters and limits used.	Appendix 2
Selection process	8	Specify the methods used to decide whether a study met the inclusion criteria of the review, including how many reviewers screened each record and each report retrieved, whether they worked independently, and if applicable, details of automation tools used in the process.	3-5
Data collection	9	Specify the methods used to collect data from reports, including how many reviewers collected data from each report, whether they worked	3-5

Section and Topic	Item #	Checklist item	Location where item is reported
process		independently, any processes for obtaining or confirming data from study investigators, and if applicable, details of automation tools used in the process.	
Data items	10a	List and define all outcomes for which data were sought. Specify whether all results that were compatible with each outcome domain in each study were sought (e.g. for all measures, time points, analyses), and if not, the methods used to decide which results to collect.	5, table 2 (8-11), Appendix 4
	10b	List and define all other variables for which data were sought (e.g. participant and intervention characteristics, funding sources). Describe any assumptions made about any missing or unclear information.	5, table 2 (8-11), Appendix 4
Study risk of bias assessment	11	Specify the methods used to assess risk of bias in the included studies, including details of the tool(s) used, how many reviewers assessed each study and whether they worked independently, and if applicable, details of automation tools used in the process.	4
Effect measures	12	Specify for each outcome the effect measure(s) (e.g. risk ratio, mean difference) used in the synthesis or presentation of results.	n/a
Synthesis methods	13a	Describe the processes used to decide which studies were eligible for each synthesis (e.g. tabulating the study intervention characteristics and comparing against the planned groups for each synthesis (item #5)).	4
	13b	Describe any methods required to prepare the data for presentation or synthesis, such as handling of missing summary statistics, or data conversions.	4
	13c	Describe any methods used to tabulate or visually display results of individual studies and syntheses.	3-4
	13d	Describe any methods used to synthesize results and provide a rationale for the choice(s). If meta-analysis was performed, describe the model(s), method(s) to identify the presence and extent of statistical heterogeneity, and software package(s) used.	4
	13e	Describe any methods used to explore possible causes of heterogeneity among study results (e.g. subgroup analysis, meta-regression).	n/a
	13f	Describe any sensitivity analyses conducted to assess robustness of the synthesized results.	n/a
Reporting bias assessment	14	Describe any methods used to assess risk of bias due to missing results in a synthesis (arising from reporting biases).	n/a
Certainty assessment	15	Describe any methods used to assess certainty (or confidence) in the body of evidence for an outcome.	n/a
**RESULTS**			
Study selection	16a	Describe the results of the search and selection process, from the number of records identified in the search to the number of studies included in the review, ideally using a flow diagram.	6
	16b	Cite studies that might appear to meet the inclusion criteria, but which were excluded, and explain why they were excluded.	n/a
Study characteristics	17	Cite each included study and present its characteristics.	table 2 (8-11),
Risk of bias in studies	18	Present assessments of risk of bias for each included study.	Appendix 3

Section and Topic	Item #	Checklist item	Location where item is reported
Results of individual studies	19	For all outcomes, present, for each study: (a) summary statistics for each group (where appropriate) and (b) an effect estimate and its precision (e.g. confidence/credible interval), ideally using structured tables or plots.	n/a
Results of syntheses	20a	For each synthesis, briefly summarise the characteristics and risk of bias among contributing studies.	12
	20b	Present results of all statistical syntheses conducted. If meta-analysis was done, present for each the summary estimate and its precision (e.g. confidence/credible interval) and measures of statistical heterogeneity. If comparing groups, describe the direction of the effect.	n/a
	20c	Present results of all investigations of possible causes of heterogeneity among study results.	n/a
	20d	Present results of all sensitivity analyses conducted to assess the robustness of the synthesized results.	n/a
Reporting biases	21	Present assessments of risk of bias due to missing results (arising from reporting biases) for each synthesis assessed.	n/a
Certainty of evidence	22	Present assessments of certainty (or confidence) in the body of evidence for each outcome assessed.	n/a
**DISCUSSION**			
Discussion	23a	Provide a general interpretation of the results in the context of other evidence.	14-16
	23b	Discuss any limitations of the evidence included in the review.	16
	23c	Discuss any limitations of the review processes used.	16
	23d	Discuss implications of the results for practice, policy, and future research.	17
**OTHER INFORMATION**			
Registration and protocol	24a	Provide registration information for the review, including register name and registration number, or state that the review was not registered.	2
	24b	Indicate where the review protocol can be accessed, or state that a protocol was not prepared.	2
	24c	Describe and explain any amendments to information provided at registration or in the protocol.	n/a
Support	25	Describe sources of financial or non-financial support for the review, and the role of the funders or sponsors in the review.	n/a
Competing interests	26	Declare any competing interests of review authors.	
Availability of data, code and other materials	27	Report which of the following are publicly available and where they can be found: template data collection forms; data extracted from included studies; data used for all analyses; analytic code; any other materials used in the review.	n/a

From: Page MJ, McKenzie JE, Bossuyt PM, Boutron I, Hoffmann TC, Mulrow CD, et al. The PRISMA 2020 statement: an updated guideline for reporting systematic reviews. BMJ 2021;372:n71. doi: 10.1136/bmj.n71

For more information, visit: http://www.prisma-statement.org/

APPENDIX 2: Full Search Strategy

#	Search String
**1**	Perinatal mental health OR postnatal period OR postpartum depression OR perinatal anxiety OR mothers/pregnancy/perinatal period AND mental health OR maternal mental health
**2**	Indigenous populations OR Māori OR First Nations OR Aboriginal OR Alaskan Natives OR Hawaiian Natives OR Pacific OR Pasifika OR Polynesian OR Torres Strait Island* OR Indigenous Peoples OR Native Americans OR Canadian Natives OR Indigenous Canadians
**3**	Crisis Intervention Services OR Group Intervention OR Crisis Intervention OR Family Intervention OR intervention OR Early Intervention OR Intervention OR Mental Health Services OR Health Care Delivery OR Health Care Services OR Community Services OR community MH services OR indigenous services OR Cultural Sensitivity OR indigenous workers OR kaupapa Māori OR cultural responsiveness
**4**	Pregnancy Outcomes OR Treatment Outcomes OR Health Outcomes OR Psychotherapeutic Outcomes OR Psychosocial Outcomes OR outcomes OR program evaluation OR Program Evaluation
**5**	1 AND 2
**6**	1 AND 2 AND 3
**7**	1 AND 2 AND 3 AND 4
**8**	Rongoā/Toanga Pūoro/Ceremony/Two eyed seeing/Medicine wheel /spirituality/Elders /kaumātua /aunt*/Traditional medicine/customary
**9**	8 AND 7

APPENDIX 3: CONSIDER statement assessment

CONSIDER STATEMENTS	Ginsberg	Walkup et	Zarnowiec	Barlow et	Ward et	Hiratsuka	Barlow et	Penehira,	Mullany,	Campbell,	Kohrt et	McCalman	Chomat et	Smith et	Ussher et	Barlow et	Dietsch et	Lowell et	Smith et	Blanchet-	Kandasam	Reid et al,	Abbott et	Chamberla	Ware et	Berryman	James et
**GOVERNANCE 1.** *Describe partnership agreements between the research institution and Indigenous-governing organization for the research*	0	0	0	1	0	0	0	0	1	0	0	0	0	0	0	0	0	0	0	1	1	1	0	1	0	0	0
**GOVERNANCE 2:** *Describe accountability and review mechanisms within the partnership agreement that addresses harm minimization.*	0	1	0	1	0	0	0	0	1	0	0	0	0	0	0	1	0	0	0	1	1	1	0	1	0	0	0
**GOVERNANCE 3.** *Specify how the research partnership agreement includes protection of Indigenous intellectual property and knowledge arising from the research, including financial and intellectual benefits generated*	0	0	0	0	0	0	0	0	0	0	0	0	0	0	0	0	0	0	0	0	1	1	0	0	0	0	0
**PRIORITISATION 4.** *Explain how the research aims emerged from priorities identified by either Indigenous stakeholders, governing bodies, funders, non-government organization(s), stakeholders, consumers, and empirical evidence*	0	0	0	1	1	0	0	1	1	1	1	0	1	0	0	0	0	1	0	1	1	1	0	1	1	1	0
**RELATIONSHIPS 5.** *Specify measures that adhere and honor Indigenous ethical guidelines, processes, and approvals for all relevant Indigenous stakeholders, recognizing that multiple Indigenous partners may be involved,*	0	0	0	1	1	0	0	1	1	0	0	1	1	1	0	0	0	1	1	1	1	1	1	1	1	1	1

**RELATIONSHIPS 6.** *Report how Indigenous stakeholders were involved in the research processes (i.e., research design, funding, implementation, analysis, dissemination/recruitment).*	**1**	**1**	**0**	**1**	**1**	**0**	**1**	**1**	**1**	**1**	**0**	**0**	**1**	**1**	**0**	**0**	**0**	**1**	**1**	**1**	**1**	**1**	**1**	**1**	**1**	**1**	**1**
**RELATIONSHIPS 7.** *Describe the expertise of the research team in Indigenous health and research.*	**0**	**0**	**0**	**0**	**1**	**0**	**0**	**0**	**0**	**1**	**0**	**0**	**1**	**0**	**0**	**0**	**0**	**1**	**0**	**1**	**1**	**0**	**1**	**1**	**1**	**1**	**1**
**METHODOLOGIES 8.** *Describe the methodological approach of the research including a rationale of methods used and implication for Indigenous stakeholders, e.g., privacy and confidentiality (individual and collective)*	**0**	**0**	**0**	**0**	**1**	**0**	**0**	**1**	**1**	**0**	**1**	**1**	**1**	**1**	**0**	**0**	**1**	**1**	**1**	**1**	**1**	**1**	**1**	**1**	**1**	**1**	**1**
**METHODOLOGIES 9.** *Describe how the research methodology incorporated consideration of the physical, social, economic and cultural environment of the participants and prospective participants*	**0**	**0**	**0**	**0**	**0**	**0**	**0**	**1**	**1**	**1**	**0**	**0**	**1**	**1**	**0**	**0**	**1**	**1**	**1**	**1**	**1**	**1**	**1**	**1**	**1**	**1**	**0**
**PARTICIPATION 10.** *Specify how individual and collective consent was sought to conduct future analysis on collected samples and data*	**0**	**0**	**0**	**0**	**1**	**0**	**0**	**0**	**0**	**0**	**0**	**0**	**0**	**0**	**0**	**0**	**0**	**0**	**0**	**0**	**1**	**0**	**0**	**0**	**0**	**0**	**0**
**PARTICIPATION 11.** *Described how the resource demands (current and future) placed on Indigenous participants and communities involved in the research were identified and agreed upon including any resourcing for participation, knowledge, and expertise*	**0**	**0**	**0**	**0**	**0**	**0**	**0**	**0**	**1**	**0**	**0**	**0**	**0**	**0**	**0**	**0**	**0**	**0**	**0**	**0**	**0**	**0**	**1**	**0**	**0**	**0**	**1**
**PARTICIPATION 12.** *Specify how biological tissue and other samples including data were stored, explaining*	**0**	**0**	**0**	**0**	**0**	**0**	**0**	**0**	**0**	**0**	**0**	**0**	**0**	**0**	**0**	**0**	**0**	**0**	**0**	**0**	**0**	**0**	**0**	**0**	**0**	**0**	**0**

the processes of removal from traditional lands, if done, and of disposal.																											
**CAPACITY 13.** *Explain how the research supported the development and maintenance of Indigenous research capacity (e.g., specific funding of Indigenous researchers).*	**0**	**0**	**0**	**0**	**1**	**0**	**0**	**0**	**1**	**0**	**0**	**0**	**0**	**0**	**0**	**0**	**0**	**0**	**0**	**0**	**0**	**0**	**0**	**1**	**1**	**1**	**0**
**CAPACITY 14.** *Discuss how the research team undertook professional development opportunities to develop the capacity to partner with Indigenous stakeholders?*	**0**	**0**	**0**	**0**	**1**	**0**	**0**	**0**	**1**	**0**	**0**	**0**	**0**	**0**	**0**	**0**	**0**	**0**	**0**	**0**	**0**	**0**	**0**	**0**	**0**	**0**	**0**
**ANALYSIS & INTERPRETATION 15.** *Specify how the research analysis and reporting supported critical inquiry and a strength-based approach that was inclusive of Indigenous values.*	**1**	**0**	**0**	**0**	**1**	**0**	**0**	**1**	**0**	**0**	**0**	**1**	**0**	**1**	**0**	**0**	**0**	**0**	**1**	**1**	**1**	**1**	**1**	**1**	**1**	**1**	**0**
**DISSEMINATION 16.** *Describe the dissemination of the research findings to relevant Indigenous governing bodies and peoples.*	**0**	**1**	**0**	**0**	**1**	**0**	**0**	**1**	**1**	**0**	**0**	**0**	**0**	**0**	**0**	**0**	**0**	**0**	**1**	**1**	**1**	**1**	**0**	**1**	**0**	**1**	**0**
**DISSEMINATION 17.** *Discuss the process for knowledge translation and implementation to support Indigenous advancement (e.g., research capacity, policy, investment).*	**0**	**0**	**0**	**0**	**1**	**0**	**0**	**0**	**0**	**0**	**1**	**0**	**0**	**0**	**0**	**0**	**0**	**0**	**0**	**0**	**0**	**0**	**0**	**0**	**0**	**0**	**0**
**TOTAL**	**2**	**3**	**0**	**5**	**11**	**0**	**1**	**7**	**11**	**4**	**3**	**3**	**6**	**5**	**0**	**1**	**2**	**6**	**6**	**10**	**12**	**10**	**7**	**11**	**8**	**9**	**5**

Table 5 CONSIDER Statements score

APPENDIX 4: General characteristics of included studies

STUDY	Psychoeducation	Behavioural Skills	Problem-solving skills	Social supports	Future Planning	Counselling / Therapy	Social determinants addressed	Relationships / TRUST	Number of sessions / contacts prescribed	Strengths based
Ginsburg et al, 2012	*	*	*	*	*	*	*		*	
Walkup et al, 2009		*	*			*			*	
Zarnowiecki et al, 2018				*	*		*	*	*	
Barlow et al, 2015	*	*	*		*	*		*	*	
Ward et al, 2022	*	*	*	*	*	*		*	*	*
Hiratsuka et al, 2018	*	*		*	*	*	*	*	*	*
Barlow et al, 2006					*				*	
Penehira et al, 2013		*	*	*			*	*		*
Mullany et al, 2012	*	*	*		*		*	*	*	
Campbell, 2018	*	*	*	*			*	*		*
Kohrt, 2022	*	*	*	*				*	*	
McCalman et al, 2015		*	*	*	*		*	*		*
Chomat, 2019	*	*	*	*	*		*	*	*	
Smith et al, 2007		*	*	*	*		*	*		*
Ussher et al, 2016	*	*	*	*		*		*		*
Barlow et al, 2013	*	*	*		*			*	*	
Dietsch, 2011				*				*		*
Lowell et al, 2015		*	*	*	*			*		*
Smith et al, 2006				*			*	*		*
Blanchet-Cohen et al, 2021	*	*	*	*	*	*	*	*		*

Kandasamy et al, 2017			*	*	*		*	*		*
Reid et al, 2021		*	*	*	*	*	*	*		*
Abbott et al, 2022		*	*	*	*			*		*
Chamberlain et al, 2021	*	*	*	*	*	*	*			*
Ware et al, 2015								*	N/A	*
Berryman et al, 2022				*				*	N/A	*
James et al, 2021			*	*	*		*	*		*
